# EKF–GPR-Based Fingerprint Renovation for Subset-Based Indoor Localization with Adjusted Cosine Similarity

**DOI:** 10.3390/s18010318

**Published:** 2018-01-22

**Authors:** Junhua Yang, Yong Li, Wei Cheng, Yang Liu, Chenxi Liu

**Affiliations:** School of Electronic and Information, Northwestern Polytechnical University, Xi’an 710072, China; ruikel@nwpu.edu.cn (Y.L.); pupil_119@nwpu.edu.cn (W.C.); HNliuyang90@163.com or hnliuyang90@mail.nwpu.edu.cn (Y.L.); pollux@mail.nwpu.edu.cn (C.L.)

**Keywords:** indoor localization, RSSI, fingerprint, Extended Kalman Filter, Gaussian Process Regression, adjusted cosine similarity

## Abstract

Received Signal Strength Indicator (RSSI) localization using fingerprint has become a prevailing approach for indoor localization. However, the fingerprint-collecting work is repetitive and time-consuming. After the original fingerprint radio map is built, it is laborious to upgrade the radio map. In this paper, we describe a Fingerprint Renovation System (FRS) based on crowdsourcing, which avoids the use of manual labour to obtain the up-to-date fingerprint status. Extended Kalman Filter (EKF) and Gaussian Process Regression (GPR) in FRS are combined to calculate the current state based on the original fingerprinting radio map. In this system, a method of subset acquisition also makes an immediate impression to reduce the huge computation caused by too many reference points (RPs). Meanwhile, adjusted cosine similarity (ACS) is employed in the online phase to solve the issue of outliers produced by cosine similarity. Both experiments and analytical simulation in a real Wireless Fidelity (Wi-Fi) environment indicate the usefulness of our system to significant performance improvements. The results show that FRS improves the accuracy by 19.6% in the surveyed area compared to the radio map un-renovated. Moreover, the proposed subset algorithm can bring less computation.

## 1. Introduction

Localization and tracking play an important role in many military, industry, transportation, and tourism applications, including tracking airplanes, positioning a new product in the workshop, tracking the behavior of wild, protected animals, positioning firefighters inside a burning building, or guiding visitors to find the right track. The most popular and familiar localization method, as we all know, is the Global Positioning System (GPS). With GPS chips being embedded in many personal devices such as smartphones, sports watches, and traffic navigators, GPS localization has been proven very suitable for consumers. Through the Global Navigation Satellite System (GNSS), GPS system provides timing data to localization devices from multiple satellites. The device can trilaterate its position on the earth by using this data. The GPS system works well in clear view environments or open spaces, but encounters a problem in indoor environments where the signal suffers from non-line-of-sight (NLOS) propagation. In this case, indoor localization technology gradually developed.

An indoor localization system is a framework consisting of a network of customized or commercial devices that are used to wirelessly locate people or objects inside an indoor environment. For localization purposes, some technologies require extensive and additional deployment of infrastructure covering the service area individually [1,2,3,4]. They may take much manpower and material resources on specialized client devices or tags attached to an object. Relatively speaking, the superiority of Received Signal Strength Indicator (RSSI)-based Wireless Local Area Networks (WLAN) positioning is embodied incisively and vividly [5,6]. There are two options for RSSI-based positioning: one option is to exploit the Access Points (APs) locations and the radio propagation path-loss models; another is fingerprinting algorithms with building floor plans. However, since the AP locations are not usually accessible and the path-loss model is uncertain, it makes this approach less attractive. The fingerprinting method has been shown to achieve more accurate positioning results than the method based on path-loss models. It does not require any extra investment cost in deploying APs by employing schemes of Wi-Fi fingerprinting [7,8,9], i.e., widespread APs installed in the building can be used for location estimation. Not only that, but the mobile devices are also widely available nowadays and are equipped with WLAN interface so there is no need for additional hardware to change or attach.

**Figure 1:**
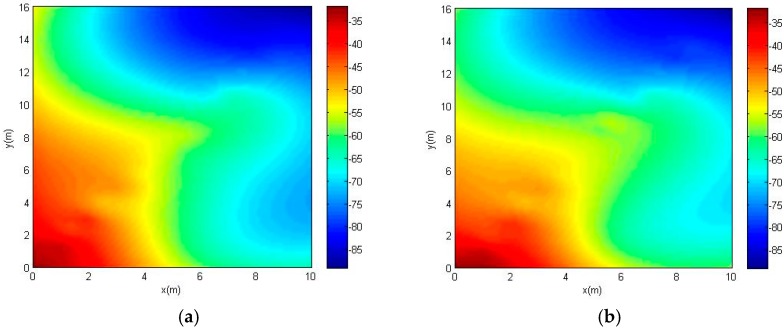
Fingerprint heat maps of one access point (AP; Media Access Control (MAC): B8-F8-83-CF-F8-96) on two different days. (**a**) 27 September 2017; (**b**) 27 October 2017.

The RSSI fingerprint-based technique, however, requires an offline phase in which RSSI measurements are collected at known locations to initial training or calibration fingerprint data. Then, the position of a target device (TD) is estimated by matching an immediate RSSI measurement with the training data in the online phase [10,11]. In the offline phase, fingerprint collection is usually labor- and time-intensive, which eventually reduces localization efficiency and increases the cost. In addition, the measured RSSI values depend on fingerprint density, positions of access points, and locations of indoor objects. The cost of these manual calibrations holds back the widespread adoption of fingerprinting indoor localization. What is more serious is that the RSSI of AP is a variable varying with time, as shown in Figure 1, the signal heat map of an AP changes (dBm) on 27 September and 27 October, 2017. The test position is in indoor environment comprised of walls, doors, furniture, and people. It is obvious that the heat maps are different in a fixed area. Consequently, a new fingerprinting updated system is required whenever the environment alters in the indoor area [12].

To address this problem, several approaches for reducing fingerprinting load or updating the fingerprinting survey radio map have been proposed. One solution is proposed in [13], which considers having an incomplete fingerprint radio map with realistic coverage gaps, and studies the performance of several interpolation methods for recovering the missing fingerprint data. In the localization framework using unsupervised manifold alignment [14], it requires very little of the fingerprinting load, some crowdsourcing-based RPs, and plan coordinates of the indoor area. Further, without building a full fingerprinted radio map of the indoor environment, a scheme tries to construct the radio map with limited calibration [15]. However, all these works just attach importance to reduce the effort of creating the radio map, but ignore the localization accuracy. There is a lack of methods to achieve an acceptable accuracy as well as reduction of the signal acquisition.

In this paper, we propose a Fingerprint Renovation System (FRS) to further reduce the fingerprinting load while still maintaining high exactness in performance. In the offline phase, we first built a fingerprinting radio map, as done traditionally. After that, the system is renovated, using a modified hybrid EKF–GPR algorithm, combining Extended Kalman Filter (EKF) and Gaussian Process Regression (GPR), where crowdsourcing-based points [16] add the localization region. In our research, a crowdsourcing-based point is defined as RP in a certain location when the original radio map needs an upgrade, and a crowdsourcing-based signal is defined as the RSSI value at the crowdsourcing-based point. In the online phase, a dynamic localization subset is detected to reduce computational cost. Then, the Weighted K Nearest Neighbor (WKNN) [17] employing ACS methods will be used for positing the TD. In order to make FRS fully limpid, we show our novel contributions as follows:

- As altered environment may be frequent in the radio map maintenance phase, we propose a novel and fast effective algorithm, which renovates the radio map by detecting the crowdsourcing-based signal. EKF–GPR is used to estimate the renovated radio map. The initial radio map is acted as state variable, and the crowdsourcing-based signal is treated as observed variable. The renovated radio map is also the state variable in the next renovation phase. In a real experiment environment, data collection works of 1030 fingerprint points are replaced by 50 crowdsourcing-based points, while the localization error is only increased by 19.2%.
- In the online phase, the calculation of matching algorithm using each fingerprint of the entire radio map is too large. In our proposed system, a specified amount of fingerprints is picked up randomly to execute the matching algorithm. The randomly subset of radio map is ***S1***. Then, a subset ***S2*** in the entire radio map is produced around fingerprints acquired by matching algorithm, and an accurate position can be acquired.
- When choosing the matching algorithm, the ACS method is presented to replace the Cosine Similarity [18]. Errors in localization results are caused by using Cosine Similarity when the target point (TP) and chosen RPs are on the same line, because Cosine Similarity only spots the differences from the vector direction. In order to correct this error, Cosine Similarity is adjusted to improve the localization accuracy in this paper.

**Figure 2:**
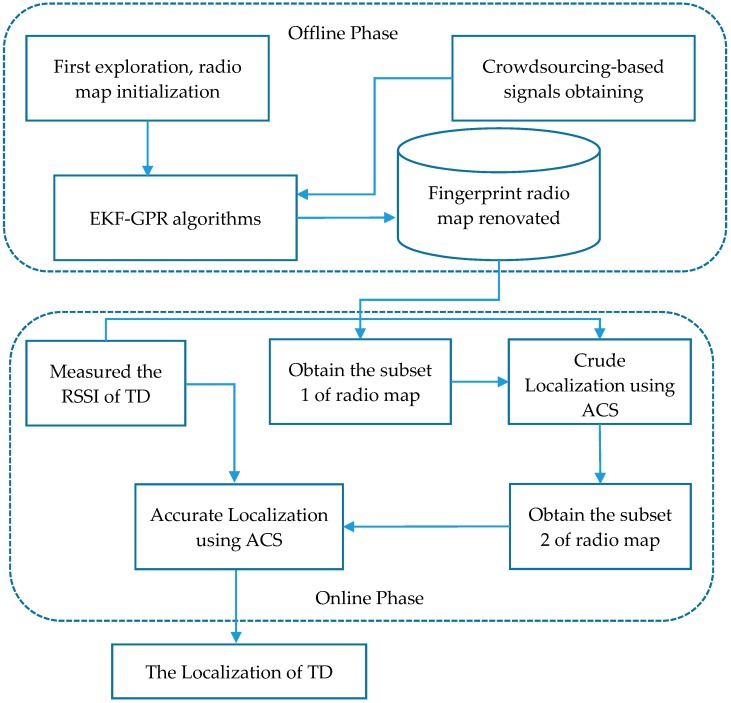
System overview of the Fingerprint Renovation System (FRS). EKF, Extended Kalman Filter; GPR, Gaussian Process Regression; RSSI, received signal strength indicator; TD, target device; ACS, Adjusted Cosine Similarity.

Figure 2 shows the framework of the proposed algorithms, which consists of the offline phase and online phase. The rest of the paper is organized as follows. We first introduce studies that relate to updating the radio map, including some major symbols, in Section 2. Section 3 proposes EKF–GPR algorithms for automatically updating the indoor positioning model. After that, we present the method of acquiring the subset in the whole fingerprinting radio map in Section 4, and ACS method is used to actualize the localization of TD in Section 5. In order to test and verify the FRS, we evaluate our methods with Wi-Fi data obtained in a real indoor environment in Section 6. Finally, we conclude this article. Though the study is in the context of Wi-Fi fingerprinting, our FRS is an independent and general method. The FRS, therefore, may be used with any fingerprint signal, such as Bluetooth, Near Field Communication (NFC), cellular networks, and Radio Frequency Identification (RFID).

## 2. Related Work

The RSSI fingerprint matching method is used as the basic scheme of many indoor localization systems at present. It is an infrastructure-free approach without the requirement of utilizing expensive hardware. A common practice to construct an initialization radio map is to manually collect fingerprints at numerous known locations in the entire localization site. The radio map requires to update the area of interest to provide the accuracy that meets the requirements of daily life. The fingerprinting radio map can be updated automatically using crowdsourcing and machine learning methods [19,20]. As in previous studies [21], it is assumed that volunteers turn on their WLAN modules to contribute their traces of RSSI measurements while carrying wireless devices, i.e., mobile phones, in a localization building. First of all, a fingerprinting radio map should be built as the initialization data map.

It is required to generate a RSSI radio map during the offline phase of fingerprint-based localization. The construction of the fingerprinting radio map begins by dividing the site of interest into grids, which needs to know the floor plan in advance. RSSI values of the wireless signals transmitted by APs are gathered in calibration points inside the grids and stored into the radio map. This radio map is used for estimating the user’s location during the online phase [22].

In the offline phase of two-dimensional (2-D) model, we divide the certain physical area into *R* known small cells. L={l_1,l_2,…,l_j,…,l_R} is defined as the center coordinates of these cells and:

(1) $$l\_j=\left(x_{j},y_{j}\right).$$

$l_{j}$ is modelled as reference points (RPs). RSSI values from all access points (APs) within a certain range are measured and stored in the fingerprint data base, which is a measurement of data vectors that can be shown as:

(2) $$P\left(l_{j}\right)=\left(p_{1}\left(l_{j}\right),p_{2}\left(l_{j}\right),\ldots ,p_{i}\left(l_{j}\right),\ldots ,p_{A}\left(l_{j}\right)\right),$$

where *j* = 1, 2, …, *R*, *R* is the number of reference points(RPs), *A* is the number of APs selected in the localization area, $p_{i}\left(l_{j}\right)$ is the RSSI value from the *i*-th AP at RP $l_{j}$. The unique Media Access Control (MAC) address is used to discriminate different APs. Regularly, RSSI from many APs are detectable somewhere in a particular area, for those APs too weak to detect or under a certain value (e.g., −100 dBm) are set as $p_{i}=0$. Then, all fingerprint signals in the radio map are:

(3) $$P=\left(P\left(l_{1}\right),P\left(l_{2}\right),\ldots ,P\left(l_{j}\right),\ldots ,P\left(l_{R}\right)\right).$$

In the online phase, the RSSI of the target is surveyed as:

(4) $$P^{T}=\left(p_{1}^{T},p_{2}^{T},\ldots ,p_{i}^{T},\ldots p_{A}^{T}\right).$$

The important notations are listed in Table 1. The radio map can be modified or updated before applying it in the online phase. In our system, it will be freshened with the help of crowdsourcing and EKF–GPR algorithms.

## 3. EKF–GPR Algorithms

The Kalman Filter has been associated with indoor localization in numerous researches. However, the function of the Kalman Filter in these research is, more often than not, concentrated upon path prediction and navigation [23,24,25,26]. There are few studies about using the Kalman Filter in fingerprint radio map updating, especially in combination with GPR. For all we know, GPR is used to update the radio map in [27] and estimate the virtual RPs’ RSSI values in [28], but it is only GPR. On the other hand, the predictive capabilities are limited by the noise covariance and uncertainty of system model in classical filter algorithms. To solve this problem, GPR is used to provide the uncertainty of predictive value in combining EKF. The EKF–GPR algorithms do not depend on observation model and parametric prediction. Using non-parametric regression, all their parameters can be learned from training data.

### 3.1. EKF in Fingerprint Radio Map Renovation

In our proposed system, the primary method used in renovating the fingerprint is EKF. The EKF iteratively estimates the fingerprint radio map and updates the estimate with crowdsourcing-based points. In the renovation process, the crowdsourcing-based signal measurement equation is represented as a nonlinear model, and therefore, linearization should be performed to derive a linear equation. The EKF deals with the real-time linearization of the system function at the previous state estimate, and also includes the real-time linearization of the observation function at the corresponding predicted RSSI.

We model the localization state equation as a noisy measurement given by

(5) $$l_{j}^{k}=F^{k-1}l_{j}^{k-1}+w^{k-1}\;,$$

where the superscript *k* is the index for the discrete time sequence, $w^{k}$ is assumed to be white Gaussian noise (AWGN) with a normal probability distribution of mean 0 and variance $Q^{k}$, $w^{k}\sim N\left(0,\;Q^{k}\right)$.

When we get the crowdsourcing-based signals, the model is defined as:

(6) $$p_{i}\left(l_{j}\right)=f_{i}\left(l_{j}\right)+v\;,$$

where $f_{i}\left(l_{j}\right)$ is the noiseless RSSI value at location $l_{j}$ from AP *i*, and v is the measurement noise which is modeled as additive Gaussian noise and $v\sim N\left(0,d_{j}^{2}\right)$. In crowdsourcing-based schemes, the input locations $l_{j}$ of clients also contain uncertainty due to localization errors. Therefore, we consider beyond the standard Equation (6) the input location with error $n_{j}$:

(7) $$l_{j}=\tilde{l_{j}}+n_{j},$$

where $\tilde{l_{j}}$ is the actual 2-D location of crowdsourcing-based point, $n_{j}\sim N\left(0,\;M_{j}\right)$ The diagonal matrix $M_{j}$ is 2-by-2 size assuming each dimension is independent, that is, $M_{j}\left[j,\;j\right]=s_{j}^{2}$ where $s_{j}$ is the uncertainty of input location $l_{j}$, and all the non-diagonal elements of matrix $M_{j}$ are zero. Then, we can achieve the equation from (6) and (7):

(8) $$p_{i}\left(l_{j}\right)=f_{i}\left(\tilde{l_{j}}+n_{j}\right)+v.$$

Taylor approximation [29] using noisy input $l_{j}$:

(9) $$p_{i}\left(l_{j}\right)=f_{i}\left(l_{j}\right)+n_{j}^{T}\partial f_{i}+v,$$

where $\partial f_{i}=\partial f_{i}\left(l_{j}\right)/\partial l_{j}$ is the derivative of function $f_{i}\left(\cdot \right)$ with regard to $l_{j}$. The measurement equation in time sequence *k*, therefore, can be modeled as:

(10) $$p_{j}^{k}=H^{k}l_{j}^{k}+n_{j}^{T}\partial f_{i}^{k}+v^{k}\cdot$$

The measurement noise is additive Gaussian noise and $v^{k}\sim N\left(0,D^{k}\right)$. Measurement covariance matrix $D^{k}$ is diagonal. Table 2 summarizes the EKF processing.

The key components of an EKF are the state prediction and measurement models, which probabilistically describe the fingerprint evolution and the measurements returned by the crowdsourcing-based signals, respectively. As show in Table 2, these models are parametric descriptions of the radio map updating. The noise components and parameters of the models can be estimated from more than one crowdsourcing-based signal. Even though such EKF models are very efficient, their updating capabilities are limited because they often ignore the model aspects of the radio map renovation process.

### 3.2. GPR in Fingerprint Radio Map Renovation

GPR is an impressive, non-parametric method for learning regression functions from sample data. The fundamental advantages of GPR are their ability to learn noise and smoothness parameters from training data, their ability to provide uncertainty estimates, and their modeling flexibility [30].

In our proposed system, GPR is designed as an enhancement of measurement models and parametric prediction for EKF. In this section, we discuss the formulation of GP. The crowdsourcing-based measurement models is show in (9), then the RSSI values $p_{i}\left(l_{j}\right)$ can be rewritten as:

(11) $$p_{i}\left(l_{j}\right)=f_{i}\left(l_{j}\right)+v_{e},$$

where $v_{e}$ is additive Gaussian noise and $v_{e}\sim N\left(0,d_{j}^{2}+\partial f_{i}^{T}M_{j}\partial f_{i}\right)$. We define the covariance function $c\left(l_{*},l_{j}\right)$, which represents the correlation of two RSSIs at input locations $l_{*}$ and the RP $l_{j}$. In that way, the transfer function between location $l_{*}$ of the crowdsourcing-based point and its RSSI is defined as a Gaussian process:

(12) $$f\left(l_{*}\right)\sim G\left(m\left(l_{*}\right),c\left(l_{*},l_{j}\right)\right),$$

where $m\left(l_{*}\right)$ is mean value, and $c\left(l_{*},l_{j}\right)$ is the covariance. The covariance of RSSI can be expressed from [31]:

(13) $$cov\left(p_{a},p_{b}\right)=c\left(l_{a},l_{b}\right)+d_{j}^{2}\beta_{ab},$$

where $l_{a}$ and $l_{b}$ denote any two localizations, $\beta_{ab}=1$ if *a* = *b*, and 0 otherwise. As shown in Section 2, ***L*** is an *R*-by-2 matrix. Corresponding to the locations ***L***, $p_{c}$ is the vector of crowdsourcing-based RSSIs. Then the covariance matrix of $p_{c}$ is shown:

(14) $$cov\left(p_{c}\right)=P_{f}+d_{j}^{2}I.$$

Here, $P_{f}$ is the *R*-by-*R* covariance matrix over all *R* input RSSIs and ***I*** is the identity matrix of size *R*. All input RSSI values are jointly Gaussian process, $p\left(l\right)\sim N\left(m\left(L\right),\;P_{f}+d_{j}^{2}I\right)$, where m(L) is a vector of *R* mean RSSIs about all locations in ***L***. Based on (11) and (13), then the predicted mean RSSI $m\left(l^{k+1}\right)$ is given by

(15) $$m\left(l^{k+1}\right)=m\left(l^{k}\right)+c{\left(l^{k},L\right)}^{T}\left(p\left(l\right)-m\left(L\right)\right){\left[P_{f}+d_{j}^{2}I+diag\left\{\Gamma_{f}M_{j}\Gamma_{f}^{T}\right\}\right]}^{-1},$$

where *diag{·}* is a diagonal matrix expression, $\Gamma_{f}$ is an *R*-by-2 matrix, it contains *R* derivative function values about $\partial f_{i}$. In that way, the predictive variance of the RSSI is given by

(16) $${\left(d_{j}^{2}\right)}^{k+1}=c\left(l^{k},l^{k}\right)-c{\left(l^{k},L\right)}^{T}{\left[P_{f}+d_{j}^{2}I+diag\left\{\Gamma_{f}M_{j}\Gamma_{f}^{T}\right\}\right]}^{-1}c\left(l^{k},L\right).$$

So far, the evolution of the dynamic fingerprinting radio map is represented by the Gaussian process alone. GPR expresses relationship between the crowdsourcing-based signals and next state of radio map.

### 3.3. EKF–GPR Algorithm in FRS

**Figure 3:**
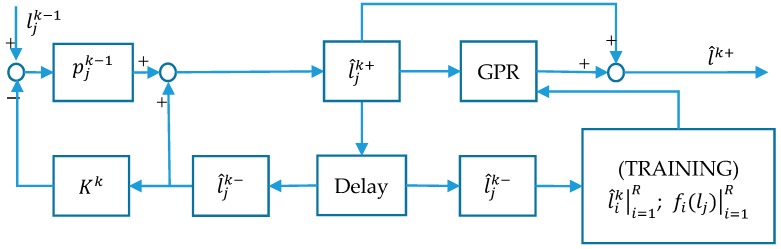
Block diagram of EKF–GPR algorithm.

Extended Kalman Filters and Gaussian Process Regression are used in conjunction to create the EKF–GPR algorithm. The block diagram of the EKF–GPR algorithm is shown in Figure 3. The EKF–GPR algorithm first employs EKF to predict the renovation result of the radio map and then uses GPR to correct and estimate the error of the EKF prediction. As we all know, there is a certain prediction error in the EKF because of the uncertainty. The prediction error is associated with state estimation to excavate the correlation in EKF–GPR algorithm.

We first use the GPR prediction model to generate the predicted mean:

(17) $${\hat{m}}^{k}=m^{k-1}+GPR_{m}\left(m^{k-1},\hat{P}\right).$$

Conditioned on observation training data ***P***, as shown in (3), the $GPR_{m}\left(\cdot \right)$ defines a Gaussian predictive distribution, and $\hat{P}$ is prediction data. Then, the input location error $n_{j}$ in (7) can be transformed:

(18) $$n_{j}=GPR_{m}\left(m^{k-1},\hat{P}\right),$$

where $n_{j}$ corresponds directly to the *GPR* uncertainty. The linearization of the prediction model is shown as:

(19) $$\Gamma^{k}=I+\frac{\partial GPR_{m}\left(m^{k-1},\hat{P}\right)}{\partial l_{j}^{k-1}}.$$

It is the sum of the identity matrix and the partial derivative of the GPR mean function. A change in any of the fingerprinting radio maps of the previous state has a direct effect on the renovation state. Meanwhile the GPR mean function only represents the change in time domain, and therefore the identity matrix is necessary. As shown step (3) of Table 2, estimation error covariance is achieved using these matrices:

(20) $${\hat{c}}^{k}=\Gamma^{k}c^{k-1}{\left(\Gamma^{k}\right)}^{T}+n_{j}.$$

According the GPR model, the predicted observation and the error covariance are shown as

(21) $$p_{j}^{k}=GPR_{m}\left(m^{k},P\right)$$

(22) $$R^{k}=GPR_{c}\left(m^{k},P\right).$$

Then the linearization of the observation model is:

(23) $$H^{k}=\frac{\partial GPR_{m}\left(m^{k},P\right)}{\partial l_{j}^{k}}.$$

From above, we can obtain the Kalman gain:

(24) $$K^{k}={\hat{c}}^{k}{\left(H^{k}\right)}^{T}{\left(H^{k}{\hat{c}}^{k}{\left(H^{k}\right)}^{T}+R^{k}\right)}^{-1}.$$

The update of the mean and estimation error covariance:

(25) $$m^{k}={\hat{m}}^{k}+K^{k}\left(p_{j}^{k}-{\hat{p}}_{j}^{k}\right)$$

(26) $$c^{k}=\left(I-K^{k}H^{k}\right){\hat{c}}^{k}.$$

Given the verified EKF model, for each crowdsourcing-based signal, EKF–GPR calculates at each RP *j* the predicted signal mean (25) and RSSI estimation error covariance (26). The EKF–GPR algorithm employs the prediction model $\Gamma^{k}$ as the input vector and the prediction errors mean $m^{k}$ as the corresponding output in each training signal. As shown in Figure 3, we train the GPR over a set of training data ${\hat{l}}_{i}^{k}$ and $f_{i}\left(l_{j}\right)$. Note that EKF–GPR utilizes a model-based EKF and a standard GPR by combining them in a cascade form.

## 4. Subset of Radio Map

Radio map is generally built in a special server, with enough hardware resource that makes it unhindered to renovate itself. In the online phase, the resource of handheld devices is constrained. The complexity of the localization algorithm is usually proportionate to the localization accuracy. On condition that the localization algorithm is confirmed, the subset of radio map using for locating should be as concise as possible. On the other hand, for the sake of preserving user privacy and to make the location system scalable, the localization algorithm should be run on the mobile unit. Hence, the scale of the radio map need to be considered. In this section, a way of subset, which runs on the final localization unit, is presented to reduce the complexity of algorithm.

In order to reduce the computation complexity, choosing only a subset of the radio map for the positioning is a convenient way. However, the subset selection usually comes at the expense of a complexity algorithm, such as machine learning techniques [32] or principal component analysis [33].

These methods bring difficulties to the localization themselves. Two stages of subset are proposed in our method to solve this issue.

In the first stage, a random subset ***S1*** is obtained from the whole radio map. Before that, the elements of matrix ***P*** in (3) is randomly sequenced to form a new matrix $P_{r}$:

(27) $$P_{r}=\left(P\left(l_{r1}\right),P\left(l_{r2}\right),\ldots ,P\left(l_{rR}\right)\right).$$

The idea of ***S1*** selection can be expressed in a mathematical form as follows:

(28) $${\left(\begin{array}{c} \begin{array}{c} P_{S1}\left(l_{r1}\right)\\ P_{S1}\left(l_{r2}\right) \end{array}\\ \begin{array}{c} \vdots \\ P_{S1}\left(l_{rM}\right) \end{array} \end{array}\right)}_{M*1}={\left(\begin{array}{ccccc} 1 & 0 & \cdots & \cdots & 0\\ 0 & 1 & \cdots & \cdots & 0\\ \vdots & \vdots & \vdots & \vdots & \vdots \\ 0 & \cdots & 1 & \cdots & 0 \end{array}\right)}_{M*R}{\left(\begin{array}{c} P\left(l_{r1}\right)\\ P\left(l_{r2}\right)\\ \vdots \\ P\left(l_{rR}\right) \end{array}\right)}_{R*1}$$

where $S1={\left[P_{S1}\left(l_{r1}\right),P_{S1}\left(l_{r2}\right),\ldots ,P_{S1}\left(l_{rM}\right)\right]}^{T}$ is subset 1 of radio map, M is the number of access points in subset 1, and M≤R, *R* is still the number of RPs in initializing radio map. In (28), the first M RPs among out-of-order array $\left\{P\left(l_{r1}\right),P\left(l_{r2}\right),\cdots ,P\left(l_{rR}\right)\right\}$ are chosen, hence,

(29) $$\left\{P_{S1}\left(l_{r1}\right),P_{S1}\left(l_{r2}\right),\ldots ,P_{S1}\left(l_{rM}\right)\right\}=\left\{P\left(l_{r1}\right),P\left(l_{r2}\right),\cdots ,P\left(l_{rM}\right)\right\}$$

can be obtained.

**Figure 4:**
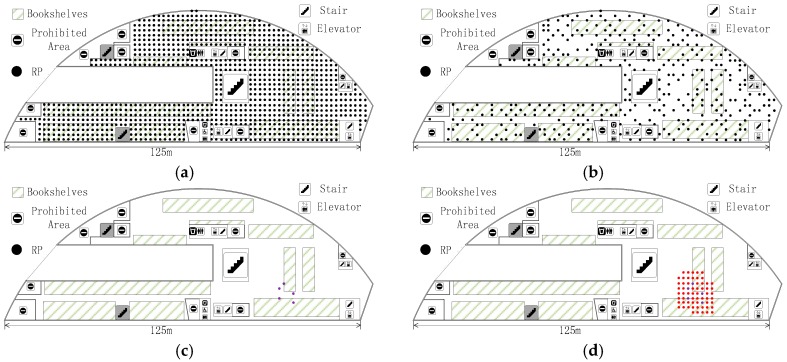
The process of choosing radio map subset. (**a**) The original reference points (RPs) in an indoor environment, which are shown as black circle dots; (**b**) Subset ***S1*** is shown as purple circle dots; (**c**) In the online phrase, five localization RPs in ***S1***; (**d**) Subset ***S2*** is shown as red triangles.

An appropriate localization algorithm, ACS in our method, is run based on ***S1***. The result is a certain amount RPs from ***S1***, which can be expressed as

(30) $$S=\left(P\left(l_{S1}\right),P\left(l_{S2}\right),\ldots ,P\left(l_{SN}\right)\right),$$

where N, N≤M, is the number of the nearest neighbor RPs in ***S1*** and it is decided by different scenes and algorithms. Then, we revive more intensive RPs in $P_{r}$ by judging which ones are around the elements of S, which is subset 2 (***S2***). In order to illustrate the whole subset choosing process, we show it in an unsophisticated indoor environment in Figure 4. The area filled by red triangles in (d) of Figure 4 is ***S2*** and it is the final subset which is used to acquire accurate localization.

## 5. Localization Based on Adjusted Cosine Similarity

During the online phase, the RSSI value in real time at the TP is compared with radio map using certain matching algorithm, such as Euclidean Distance and Cosine Similarity [34]. The algorithm of Euclidean Distance could not deliver a high localization accuracy because some statistical regularities are ignored in Euclidean Distance, which can be estimated from the TP and fingerprint radio map. On the other hand, the two algorithms can also be combined with K Nearest Neighbor (KNN) [35] and WKNN methods which focus on the calibration or calibration-free method to deal with the RSSI value difference between the RP and the TP. The KNN algorithm uses the RSSI of TP to search for K closest matches of RPs in fingerprint, then taking the mean value of these K location coordinates. WKNN is expanded on the basis of KNN but gives different weights to these K-referenced coordinates. In this paper, Cosine Similarity and WKNN are chosen to achieve TP localization.

Traditionally, Cosine Similarity investigates the correlation of RP and TP from the view point of similarity. The expression of Cosine Similarity is shown as

(31) $$CS\left(x,y\right)=\frac{\sum_{i}x_{i}y_{i}}{\sqrt{\sum_{i}x_{i}^{2}}\sqrt{\sum_{i}y_{i}^{2}}}=\frac{\langle x,y\rangle }{\left\|x\|\;\|y\right\|}.$$

However, there are deficiencies about Cosine Similarity, in that it only spots the differences from the vector direction, but ignores the absolute value. Errors in the localization result are made when using Cosine Similarity when the TP and *K* RPs are on the same line. In order to address this concern, the ACS method is presented in this paper. The correlation between RSSI vectors $P^{j}$ and $P^{T}$ is based on ACS, i.e.,

(32) $$A_{j}=ACS\left(P^{j},P^{T}\right)=\frac{\sum_{a=1}^{A}\left(P_{a}^{j}-\bar{P^{T}}\right)\left(P_{a}^{T}-\bar{P^{T}}\right)}{\sqrt{\sum_{a=1}^{A}{\left(P_{a}^{j}-\bar{P^{T}}\right)}^{2}}\sqrt{\sum_{a=1}^{A}{\left(P_{a}^{T}-\bar{P^{T}}\right)}^{2}}},$$

where $\bar{P^{T}}$ is the average RSSI value from the all APs at the TP, and:

(33) $$\bar{P^{T}}=\frac{1}{A}\overset{A}{\underset{a=1}{\sum }}P_{a}^{T}.$$

In the KNN method, *K* coordinates of most similar RPs to TP are employed to calculate the mean value. The expression is given as follows,

(34) $$\left(x_{T},y_{T}\right)=\frac{1}{K}\overset{K}{\underset{i=1}{\sum }}\left(x_{i},y_{i}\right),$$

where $\left(x_{T},y_{T}\right)$ is the measurement localization coordinate of TP, is the coordinates of *K* RPs. Without assigning weight, KNN is the basic Nearest Neighbor algorithm. Meanwhile, it is assigned a weight to every coordinate according to the value of similarity in the WKNN method. Normally, $w_{j}$ is the weight of the *j*-th selected RP, which can be calculated as

(35) $$w_{j}=\frac{1}{E_{j}\sum_{i=1}^{k}\left(\frac{1}{E_{i}}\right)},\;j=1,2,\ldots ,K.$$

In this way, the coordinate of TP using WKNN can be shown as

(36) $$\left(x_{T},y_{T}\right)=\overset{K}{\underset{i=1}{\sum }}w_{j}\left(x_{i},y_{i}\right).$$

**Figure 5:**
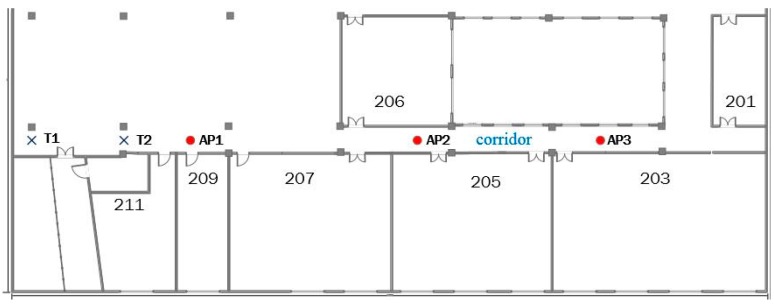
Experiment of ACS in the real environment, APs are shown as red circle dots, two test points is shown as blue crosses, and the five points are in one straight line.

In order to illustrate the improvement of ACS, we carry out an experiment in a real indoor environment. We performed the tests in the second floor of School of Electronics and Information in Northwestern Polytechnical University (NWPU). There are three APs (AP1-5c:dd:70:b6:89:71, AP2-5c:dd:70:b6:92:51, AP3-5c:dd:70:b6:75:71), that are formed in a straight line in the corridor, as shown in Figure 5. The RSSIs of T1 and T2 are shown in Table 3.

The similarity between T1 and T2 using (31) is 0.9945, and there is a remarkable homogeneity between them. If this situation is emerged in localization phrase, T1 and T2 will be judged as two very close points. However, we can see that there is a certain interval between them. Unlike the Cosine Similarity, the similarity value is 0.703 using (32). T1 and T2 are no longer that close, which meets the actual circs better.

## 6. Experimental Evaluation

In this section, we consider the experimental testbed and evaluate the performance of the proposed FRS method comparing it to other methods in the area of WLAN location determination. All systems were implemented in the NWPU library for fair comparison.

### 6.1. Experimental Settings

**Figure 6:**
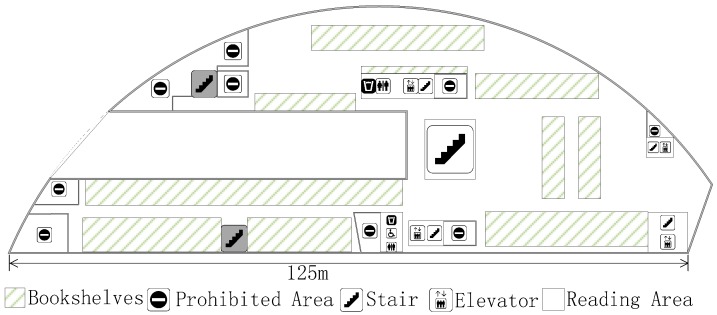
Plane graph of the fourth floor in the Northwestern Polytechnical University (NWPU) campus library.

**Figure 7:**
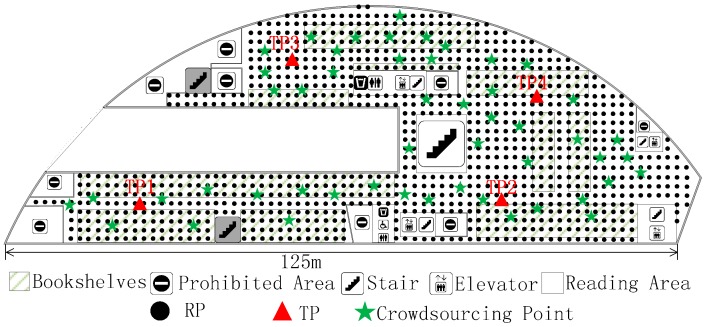
The distribution of RPs, target points (TPs), and crowdsourcing-based points in the experimental area.

The experiment is carried out at the fourth floor of the NWPU campus library. Except for the prohibited area, there is still about 4100 m^2^ left, as shown in Figure 6. The experimental area is mainly focused on bookshelves and a reading area. It has an immense space, including bookshelves, desks, and frequent flow of people. We conducted experiments on two different periods, the RPs collecting date (Database_1) was from 16 October 2017 to 22 October 2017, and the crowdsourcing-based signals were collected on 16 November 2017. The renovated radio map using crowdsourcing-based signals is marked as Database_2. In the online phase, four TPs (Figure 7) were used to detect the accuracy of localization using Database_1 and Database_2, respectively.

We collect RPs on the fourth floor of the NWPU campus library, an irregular plane, and the total number of RPs is 1030, as shown in Figure 7. These RPs were taken at two-meter intervals. All of RSSIs were taken in the middle of grids using Wi-Fi Analyzer in a Nubia Mobile Phone. Overall, 105 physical APs were detected for testing. Except for infrastructure APs, fixed on the library ceiling, there are about 10 temporary APs left. There are 10 APs between two phases and are also one part of the alternative radio map. At each RP, a client could measure 15 distinctive physical APs on average. These APs are ordered according to the RSSI value, and we choose the eight largest APs as $P\left(l_{j}\right)$. In order to obtain the exact result, we face north, west, south, and east, respectively, to sample the RSSI at every RP. In the same way, the crowdsourcing-based signal at a certain point is obtain for renovating the radio map. As we stated before, $\tilde{l_{j}}$ is the actual 2-D location of crowdsourcing-based point. In this experiment, the number of this unit is 50 and it is chosen randomly, as shown in Figure 7.

We use a number of baseline parameters in each phase. When using the WKNN localization method, the parameter *k* = 5. The number of APs at each RP or TP, A = 10, and it includes the APs with the 10 largest RSSI value. As we stated before, the total number of RPs is 1030, R=1030, and M=100 in S1. In (30), N=5 is the same as *k*. The nearest 24 RPs are revived pointing at each of the five RP, hence there are 125 revived RPs of ***S2*** at most, because these RPs may coincide with each other.

### 6.2. The Effect of Radio Map Renovation

Here, we use four TPs during the experimental period to investigate the performance of our renovation method. We conduct the ACS localization method on targets with two fingerprint radio maps, Database_1 and Database_2.

**Figure 8:**
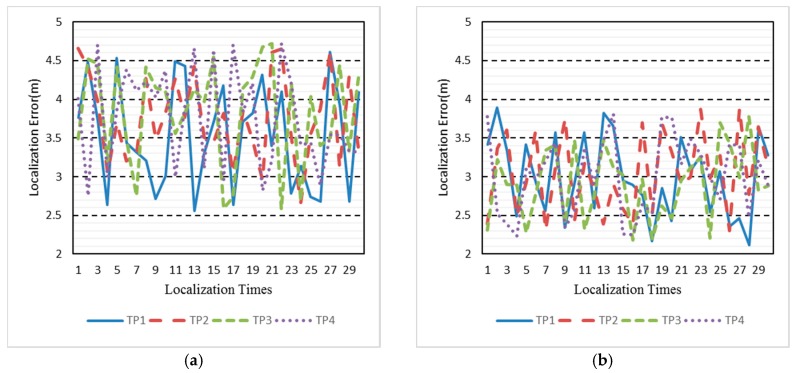
Localization error before (**a**) and after (**b**) radio map renovation.

We then evaluate localization result with the two radio maps respectively. The localization experiment is carried out on 17 November 2017, one day after radio map renovation. The four TPs are distributed in the experimental area as the red triangle, as shown in Figure 7. At each TP, the ACS localization algorithm is run 30 times with 1 min intervals. Figure 8 shows the real-time localization errors versus the 30 times location. Before renovating the radio map, as shown in Figure 8a, four TPs have similar localization errors. Given Database_2 in Figure 8b, each localization error of TP is reduced. The mean error are 3.72 m and 2.99 m, corresponding to Database_1 and Database_2, respectively.

**Figure 9:**
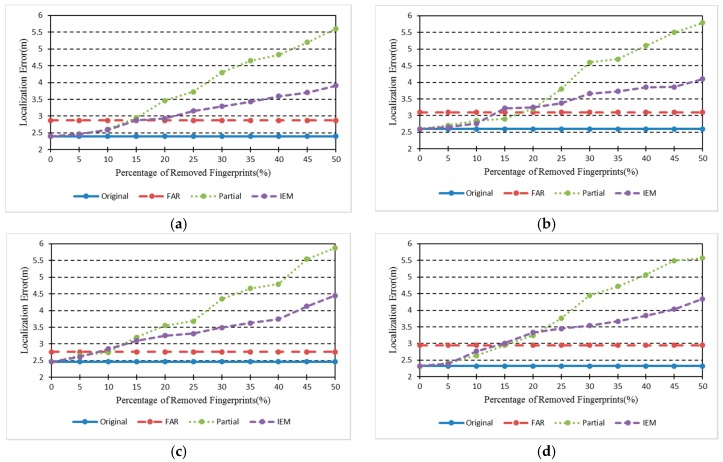
Localization results of four TPs. Original: The localization error obtained from Database_3; FAR: The localization error obtained from Database_4. Both of them are irrelative with Percentage of Removed Fingerprints. (**a**) TP1; (**b**) TP2; (**c**) TP3; (**d**) TP4.

We also compare FRS with interpolation and extrapolation methods (IEM) [13] based localization algorithms. The experimental RPs collecting date was from 28 November 2017 to 3 December 2017 and the RPs location is same as in Figure 7, which is called Database_3. Crowdsourcing-based signals are also collected during this phase to renovate Database_1. The renovated result here is Database_4, which is similar to Database_2. Then, the same four TPs shown in Figure 7 are used for localization. After that, some certain number percentages of Database_3 are removed to locate the TPs. Figure 9 shows the results of the localization using different methods and radio maps. In this Figure, *Original* is the localization result using Database_3, *FRS* represents the localization error using Database_4, *Partial* shows the localization result along with the removed percentages of Database_3, and *IEM* indicates the error using interpolation method based on different percentages of Database_3. The *IEM* method shows better localization results when the removed percentages of radio map are below 15%. However, the *FRS* method has a lower error when the removed percentages of radio map are above 15%. Although both of them reduce the labour force to update fingerprint radio map, *FRS* proposed in this paper has a greater scope of application. As the Percentage of Removed Fingerprints increases, the localization errors of *Partial* and *IEM* method grow larger. The *FRS* method is unaffected by the Removed Fingerprints and only needs 50 crowdsourcing-based points. Meanwhile, comparing *Original* and *FRS*, data collection works of 1030 fingerprint points are replaced by only 50 crowdsourcing-based points, while the mean localization error increases from 2.45 m to 2.92 m.

### 6.3. The Impact of Crowdsourcing-Based Points in Amount and Location

In the proposed FRS, the amount and position of crowdsourcing-based points directly impact the stabilization and accuracy of localization. There are 50 crowdsourcing-based points, as shown in Figure 7, to achieve all the result above. In this section, different numbers and positions of crowdsourcing-based points are set up to reveal the distinction of localization accuracy. The collection work of crowdsourcing-based signals is similar to normal RP. The only difference between the two is the randomness of crowdsourcing-based points.

**Figure 10:**
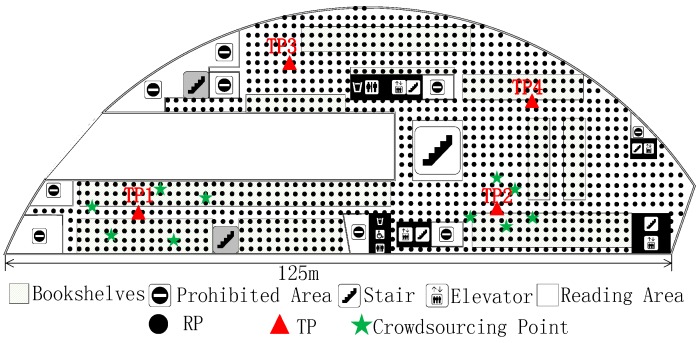
Ten crowdsourcing-based points around TP1 and TP2, away from TP3 and TP4.

**Figure 11:**
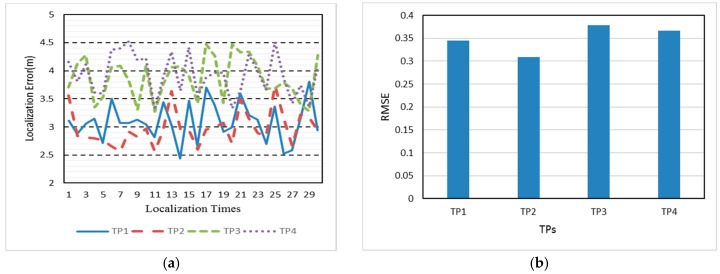
(**a**) Localization error of TP1 and TP2 compared to TP3 and TP4 with different situations about crowdsourcing-based points; (**b**) RMSEs of these four TPs.

First of all, 10 crowdsourcing-based points are set around TP1 and TP2, away from TP3 and TP4. Figure 10 shows the distribution of crowdsourcing-based points. After playing a role in the FRS system, crowdsourcing signals bring different localization accuracy as shown in Figure 11a. The mean localization error of TP1, TP2, TP3, and TP4 are 3.09 m, 2.98 m, 3.87 m, and 3.93 m, respectively. The localization errors of TP1 and TP2, which are around by crowdsourcing-based points, are reduced by 22.1% compared to TP3 and TP4. We can also see the otherness of root-mean-square error (RMSE) in Figure 11b. The localizations of TP1 and TP2 are more precise than TP3 and TP4.

**Figure 12:**
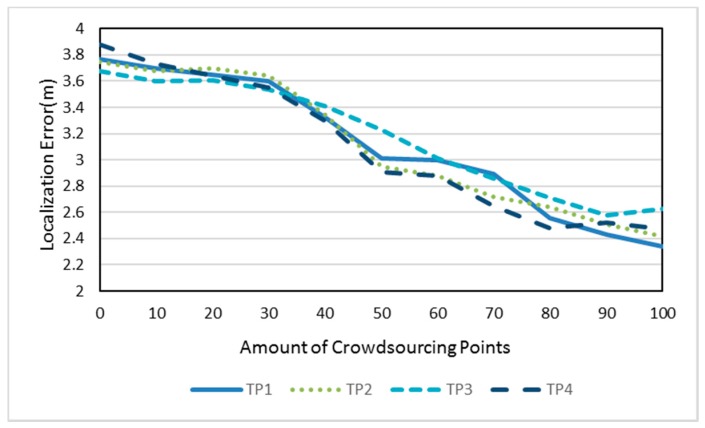
Localization error of TP1, TP2, TP3, and TP4 with different amounts of crowdsourcing-based points.

Except for positions of crowdsourcing-based points, the amount also affects the localization accuracy. In the experimental area, we added the crowdsourcing-based points randomly, as shown in Figure 7. The interval is 10 and the scope is from 0 to 100. At each TP, the localization process is repeated 30 times with different amounts of crowdsourcing-based points. The localization errors of the four TPs are shown in Figure 12.

It indicates that the localization deviation is decreasing as the amount of crowdsourcing-based points increases. The promotion of localization accuracy is obvious from 30 to 80, while the trend is slowed down at others.

## 7. Conclusions

The RSSI fingerprint technique has the advantages of simplicity, deployment practicability, and supplying reasonable accuracy. Therefore, fingerprinting localization has attracted a lot of attention. However, it is hard to upgrade a fingerprint radio map due to harsh force and intensive labor requirements. In this paper, we have proposed and tested indoor localization with FRS, a subset of a radio map, and ACS, which achieves precise indoor localization.

We tested our proposed system in a real indoor environment. For altered RSSI in different date, FRS efficiently renovated the radio map, used new fingerprint with suitable algorithms and then found the location of the TP. Compared to the mean localization 3.72 m of un-renovated radio map, we achieved 2.99 m mean error using the renovated radio map. The localization accuracy is promoted about 19.6% and this value is raising as the amount of crowdsourcing-based points increases.

Moreover, the subset choosing of radio map reduces localization computation in the proposed FRS. In our experiment, it needs repeating ACS of a total of 1030 times without subset. This number is only 125 for each TP and the computation reduced about 87% in the online phase. We also consider that the FRS can use the ACS algorithm introduced to improve their positioning systems.

## Figures and Tables

**Table 1:** Major Symbols Used in Radio Map.

Symbol	Meaning
L	Cartesian coordinates of RPs
$P\left(l_{j}\right)$	RSSI values from all APs at $l_{j}$ RP
P	RSSI values of all the RPs
$P_{T}$	RSSI values from all APs at target point
$p_{i}\left(l_{j}\right)$	RSSI value from the *i*-th AP at RP $l_{j}$
$\hat{P}$	The prediction RSSI values
K	The Kalman gain
*k*	The index for the discrete time sequence
$\bar{P\left(l_{j}\right)}$	Average RSSI value of $P\left(l_{j}\right)$
$\bar{P^{T}}$	Average RSSI value of $P^{T}$
S1, ***S2***	The subset of radio map

**Table 2:** A summary of the EKF processing.

State Model:	$l_{j}^{k}=F^{k-1}l_{j}^{k-1}+w^{k-1}$
Measurement Model:	$p_{j}^{k}=H^{k}l_{j}^{k}+n_{j}^{T}\partial f_{i}^{k}+v^{k}$
(1) Initial filter:	${\hat{l}}_{j}^{0+}=E\left(l_{j}^{0}\right)$
	$P^{0+}=E\left[\left(l_{j}^{0}-{\hat{l}}_{j}^{0+}\right){\left(l_{j}^{0}-{\hat{l}}_{j}^{0+}\right)}^{T}\right]$
(2) *k* = 1, *k* ++, calculate the partial differential matrix:	$F^{k-1}={\frac{\partial l_{j}^{k-1}}{\partial l_{j}}|}_{{\hat{l}}^{\left(k-1\right)+}}$
	$N^{k-1}={\frac{\partial l_{j}^{k-1}}{\partial w}|}_{{\hat{l}}^{\left(k-1\right)+}}$
(3) The update of state estimation and estimation error covariance:	$P^{k-}=F^{k-1}P^{\left(k-1\right)+}F^{\left(k-1\right)T}+N^{k-1}Q^{k-1}N^{\left(k-1\right)T}$
	${\hat{l}}_{j}^{k-}=\;F^{k-1}{\hat{l}}_{j}^{\left(k-1\right)+}$
(4) Calculate the partial differential matrix:	$H^{k}={\frac{\partial p_{j}^{k}}{\partial l_{j}}|}_{{\hat{l}}^{k-}}$
	$M^{k}={\frac{\partial p_{j}^{k}}{\partial v}|}_{{\hat{l}}^{k-}}$
(5) The measurement update of state estimation and the update estimation error covariance:	$K^{k}=P^{k-}H^{kT}{\left(H^{k}P^{k-}H^{kT}+M^{k}D^{k}M^{kT}\right)}^{-1}$
	${\hat{l}}_{j}^{k+}={\hat{l}}_{j}^{k-}+K^{k}\left[p_{j}^{k}-H^{k}{\hat{l}}_{j}^{k-}+n_{j}^{T}\partial f_{i}^{k}\right]$
	$P^{k+}=\left(I-K^{k}H^{k}\right)P^{k-}$
(6) Go to step (2)	

**Table 3:** The RSSIs of ACS test points.

	AP1	AP2	AP3
T1	−49 dBm	−55 dBm	−66 dBm
T2	−36 dBm	−49 dBm	−64 dBm
